# Precocious puberty related to Leydig cell testicular tumor: the diagnostic imaging keys

**DOI:** 10.1186/s40001-022-00692-1

**Published:** 2022-05-12

**Authors:** Téodor Grand, Anne-Laure Hermann, Maxime Gérard, Emmanuel Arama, Linda Ouerd, Nada Garrouche, Laurence Rocher

**Affiliations:** 1 Service de Radiologie, APHP Hôpitaux Paris Saclay, Hôpital Antoine Béclère, 157 rue de la porte de trivaux, 92140 Clamart, France; 2 Service de Radiopédiatrie, Armand Trousseau Hospital, Paris, France; 3 Service de Pédiatrie, Armand Trousseau Hospital, Paris, France; 4 Université Paris Saclay, 63 rue Gabriel Péri, 94270 Le Kremlin-Bicêtre, France; 5 BIOMAPS, IR4M, UMR8081, 4, place du Général Leclerc, 91401 Orsay cedex, France

**Keywords:** Leydig cell tumors, Testicular tumor, Precocious puberty, Shear-wave elastography, Diffusion MRI, Contrast-enhanced MRI

## Abstract

**Background:**

We report the challenging case of a 6-year-old boy with precocious puberty related to histologically proven Leydig cell tumor.

**Case presentation:**

Multiparametric ultrasound and magnetic resonance imaging (MRI) was performed. Interesting findings were scarcely or never reported in children and differed from adults Leydig cell tumors s such as the hyperechogenic halo surrounding the lesion and the dominant central vascularization using ultrasensitive Doppler. MRI revealed an enlarged testicle with strong enhancement of a tumor, a tumor apparent diffusion coefficient (ADC) of 600 × 10^−3^ mm^2^/s and a lower ADC value of the non-tumor parenchyma compared to the contralateral testis (ADC = 800 × 10^−3^ mm^2^/s vs 1100 × 10^−3^ mm^2^/s), attributed to the spermatogenesis induced by hormonal impregnation.

**Conclusion:**

We illustrate multiparametric US and MRI findings of a pediatric Leydig cell tumor, including the imaging changes attributed to local hormone secretion, which may be helpful in similar cases.

## Introduction

Precocious puberty (PP) has a major impact on the psychological and physical development and well-being of children, with an annual incidence over 0.24 per 10,000 boys [1]. Peripheral PP can be associated with testicular tumors, mostly Leydig cell tumors (LCTs). LCTs are sex cord-stromal tumors that develop from the testis stroma, and although they can occur at any age they are usually seen between 5 and 10 years of age [2]. These tumors have been reported to carry a small malignancy risk in the adult population, with no significant documented evidence of such risk in children [3], and are eligible for sparing surgery. LCTs occur in 4% of boys presenting a PP and represent 3–6% of all testicular masses found in prepubertal males [4]. Nevertheless, PP has also been described in other tumor or pseudo-tumor processes, including testicular adrenal rest tumors [5], and very rarely granulosa cell tumors [6], and germ cell tumors [7], with significant differences regarding treatment. The diagnosis and therefore management may be challenging. LCT characterization has improved in recent years in adults, however, in children US and MRI findings are scarce. With this case report, our aim is to highlight significant radiological findings in LCTs, which can help in the diagnostic process.

## Case report

**Fig. 1:**
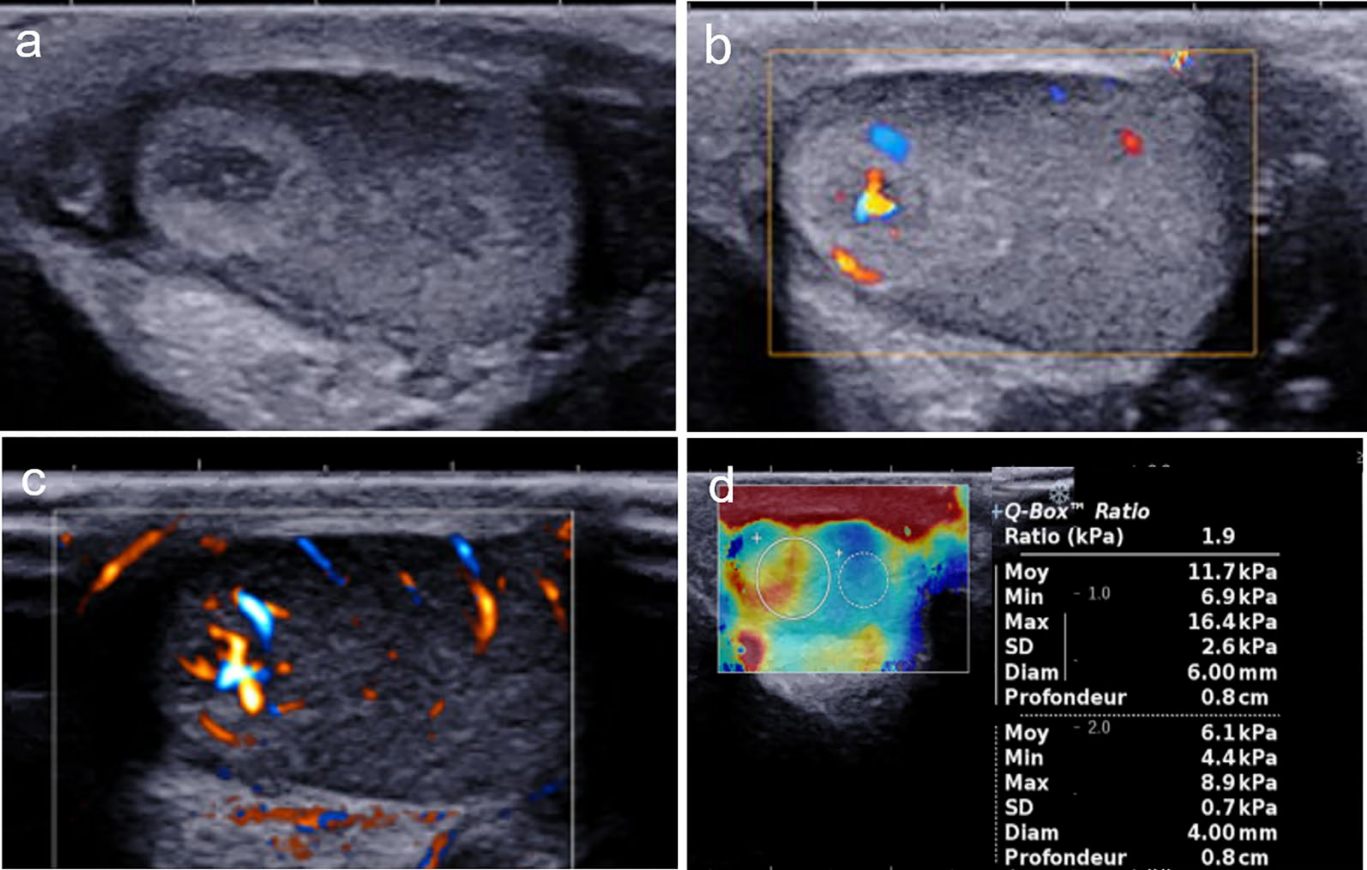
Multiparametric ultrasound. Ultrasonography of the left testicle: the tumor appeared as a hypoechoic mass surrounded by a hyperechoic rim in B-mode ultrasound (**a**), presenting predominant central vascularization in color Doppler (**b**) and ultrasensitive Doppler (**c**). Using shear-wave elastography, the tumor exhibited a moderate high stiffness (11.7 kPa) compared the adjacent parenchyma (6.1 kPa). Or right testicle (5.4 kPa, not shown)

We report the case of a 6-year-old boy presenting with advanced pubarche associated with left testicular swelling. First symptoms were observed a year prior to diagnosis by the patient’s mother, who noticed the appearance of pubic hair on the left side of the root of the penis. Levels of tumor markers, including human chorionic gonadotropin, alpha fetoprotein, and lactate dehydrogenase, were normal. Follicle-stimulating hormone, luteinizing hormone, adrenocorticotropic hormone, and 17α-hydroxyprogesterone levels were normal. Testosterone level was 0.8 nmol/mL (considered as upper limit of normal), and estradiol level was slightly increased (50 pmol/mL, *N* < 35 pmol/mL). US examination (Mach 30, Supersonic Imagine, Aix-en-Provence) showed an enlarged left testicle (estimated volume: 1.5 mL vs 0.6 mL on the right side), with a nodular ovoid-shaped hypoechoic lesion at its upper pole. The tumor exhibited a peripheral hyperechoic ring, with smooth margins and a shaped demarcation with adjacent pulp, the whole measuring 7.4 × 6 mm. color Doppler showed a hypervascular tumor compared to the surrounding tissue, with predominant central vascularization at ultrasensitive Doppler. Shear-wave elastography of the tumor, surrounding parenchyma, and contralateral testis indicated a mean stiffness of 11.7 kPa, 6.1 kPa, and 5.4 kPa, respectively (Fig. 1).

**Fig. 2:**
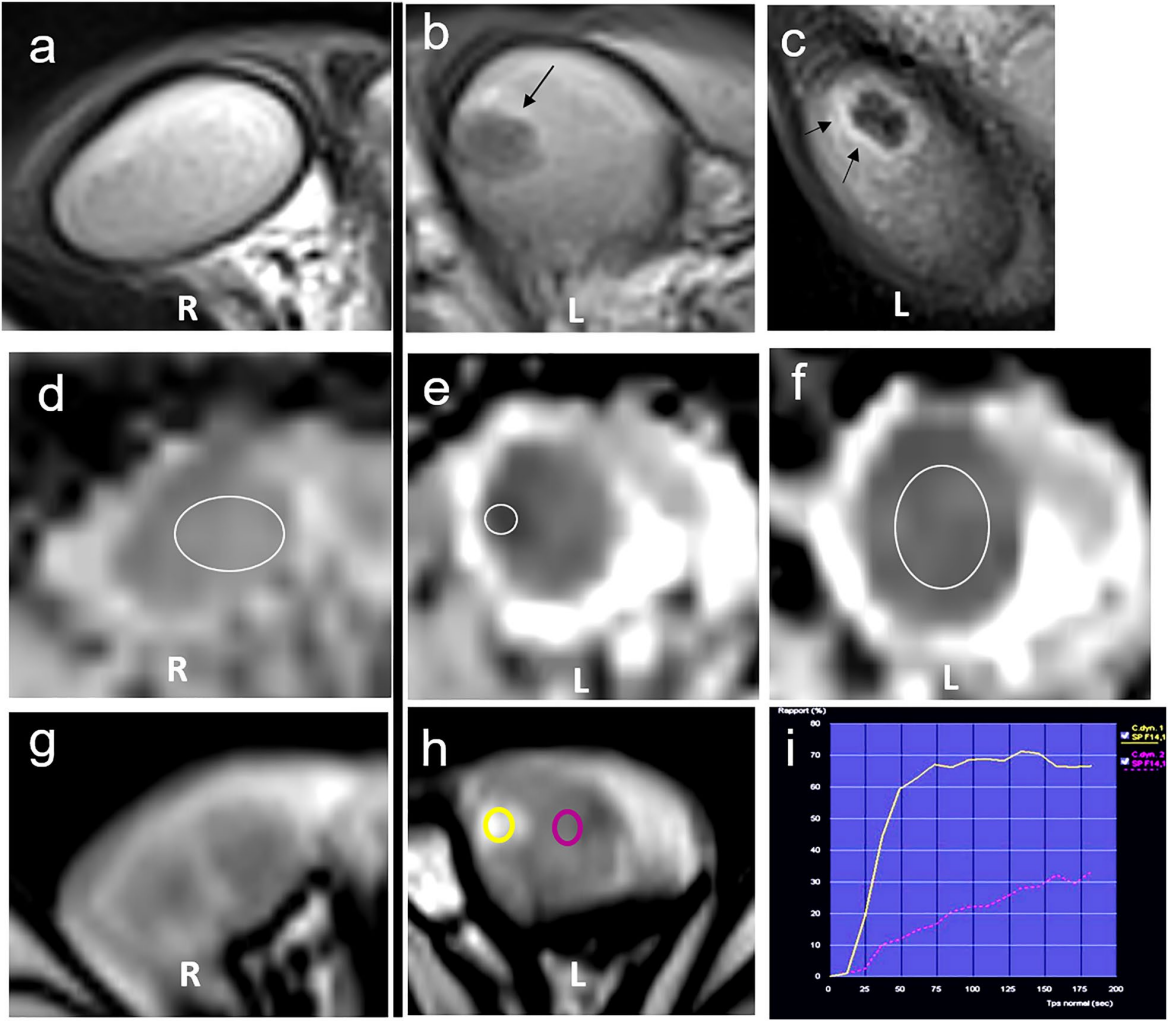
Multiparametric MRI. **a**–**c **T2-weighted sequence. Axial T2-weighted sequence showing the right homogenous testicle. **b**, **c** Axial and sagittal T2-weighted sequence shows a low-intensity, well-delimited mass with a thin high-intensity rim (left side, arrows). **d–f **ADC map **d** right testicle, ADC value than the contralateral testis = 1100 × 10^−3^ mm^2^. **e,** **f** left testicle, tumor ADC value = 600 × 10^−3^ mm^2^/s, surrounding parenchyma ADC value = 800 × 10^−3^ mm^2^. **g–i **T1 dynamic enhanced sequence. The early phase of the dynamic contrast-enhanced sequence of the right (**g**) and the left (**h**) testis showing that the tumor presented early homogeneous enhancement, and intense than the adjacent parenchyma, with a type 2 time–signal intensity curve (**f**)

Scrotal MRI using a 1.5-T scanner and a surface coil indicated that the tumor had a low-intensity signal on a T2-weighted image (with a peripheral hyperintense rim) and an iso-intensity signal on a T1-weighted image. Fast, strong and homogeneous enhancement was observed after dynamic gadolinium injection with a type 2 time–signal intensity curve without any significant wash-out, compared to the normal contralateral testis. MRI also showed slightly restricted diffusion on high *b*-value diffusion-weighted sequences (*b* = 1000 s/mm^2^) and hypo-intensity on apparent diffusion coefficient (ADC) maps (ADC = 600 × 10^−3^ mm^2^/s) (Fig. 2). The overall ADC of the parenchyma surrounding the tumor was low compared to the contralateral testis (ADC = 800 × 10^−3^ mm^2^/s vs 1100 × 10^−3^ mm^2^/s). No intraoperative frozen section was performed as lesion size was inferior to 7 mm after surgical removal. [8]. No significant postoperative complication was reported. The pathology report confirmed a 6 mm LCT, with clear surgical margins. Interestingly, an area of increased spermatogenesis around the lesion was also noted. After the procedure, plasmatic testosterone level was measured at 0.07 nmol/mL (vs 0.8 preoperatively). Clinical features remained stable during follow-up, without further increase in hair growth or new sign of precocious puberty.

## Discussion

LCTs are rare. Typical US findings in adults have been described as an isolated hypoechoic well-delimited mass with predominant peripheral hypervascularization [9]. In adults, LCTs may be discovered because of gynecomastia, but are mostly depicted an incidental finding during US infertility screening. Dynamic enhanced MRI were more likely to show faster, higher and stronger enhancement compared to normal testis or seminomatous germ cell tumors [10], similar as our pediatric case.

Testicular adrenal rests tumors (TART) described in congenital adrenal hyperplasia is the main differential diagnosis in case of PP associated with testicular mass. TARTs may be hypoechoic or hyperechoic at US, mainly located near the mediastinum and bilateral, and showing a multilocular pattern, and those signs helped to differentiate them from LCTs [10]. Their vascularization may vary, described as intense [10] or poor [11] as reported in recent case series. Depending of the scan and the color Doppler parameters. Our pediatric presentation of LCT showed interesting features, especially predominant central vascularization on ultrasensitive-Doppler, and this pattern differed from sporadic LCTs described in adults. A hyperechoic rim surrounding the tumor was also observed in our case. This feature is consistent with another recent case series [2] and has not been described in TARTs. The moderate increase of stiffness in the lesion on SWE is common in benign testicular tumors and is not useful to distinguish LCT from TART. Moreover, TARTs are usually bilateral lesions. The unilateral location of the tumor was therefore taken into account in the diagnostic process. MRI emerged as an additional diagnostic modality for the assessment of testicular mass when the US findings are equivocal in adults. MRI findings may support the initial hypothesis of LCT, showing a hypervascular tumor with early enhancement and a type 2 time–signal intensity curve, with a high, strong enhancement concordant with the previously published adult cases [10]. The overall low ADC of the left testicle is an unusual finding. Given that LCTs are known to increase intra-testicular testosterone levels and precocious spermatogenesis, we hypothesized that local hypercellularity could be an indirect manifestation of hormonal secretion in this patient, as described at pathological analysis [12].

To conclude, the predominant central vascularization on ultrasensitive-Doppler and the hyperechoic rim were the two major features to discriminate an LCT from a unilateral TART on US scan. On MRI, both the enhancement kinetic curve and the low ADC of the lesion as well as the testicular parenchyma helped to cement our preoperative hypothesis.

Diagnostic management of PP related to testicular mass may be challenging. This case illustrates the contributions brought by multiparametric US and MRI findings, including imaging changes caused by local hormone secretion, which may be helpful to distinguish an LCT from a malignant tumor or a TART, the two main differential diagnoses in PP.

## Data Availability

The data that support the findings of this study are available from the corresponding author upon reasonable request.

## Abbreviations

LCT: Leydig cell tumor
PP: Precocious puberty
US: Ultrasound
SWE: Shear-wave elastography

## References

[1] Le Moal J, Rigou A, Le Tertre A, De Crouy-Channel P, Léger J, Carel J-C (2018). Marked geographic patterns in the incidence of idiopathic central precocious puberty: a nationwide study in France. Eur J Endocrinol.

[2] Karmazyn B, Weatherly DL, Lehnert SJ, Cain MP, Fan R, Jennings SG (2018). Characteristics of testicular tumors in prepubertal children (age 5–12 years). J Pediatr Urol.

[3] Farkas LM, Székely JG, Pusztai C, Baki M (2000). High frequency of metastatic Leydig cell testicular tumours. Oncology.

[4] Trahmono null, Wahyudi I, Rodjani A, Situmorang GR, Marzuki NS.  (2020). Precocious pseudo-puberty with testicular enlargement: two cases of Leydig cell tumor with different histopathological results. Res Rep Urol..

[5] Faizah M, Zuhanis A, Rahmah R, Raja A, Wu L, Dayang A, et al. Precocious puberty in children: a review of imaging findings. Biomed Imaging Interv J. 2012 https://www.ncbi.nlm.nih.gov/pmc/articles/PMC3432225/. Accessed 5 Apr 2021. 10.2349/biij.8.1.e6 PMC3432225 22970062

[6] Antón L, Pérez-Etchepare E, Soriano D, Gómez M, Barrientos G, Tracchia R (2010). Testicular tumors: wide spectrum in our short casuistics.. Cirugia Pediatr Organo Of Soc Espanola Cirugia Pediatr..

[7] Senniappan S, Wood D, Hakeem V, Stoneham S, Freeman A, Dattani M (2014). Gonadotrophin-independent precocious puberty associated with later diagnosis of testicular embryonal carcinoma. Horm Res Paediatr.

[8] Galosi AB, Fulvi P, Fabiani A, Servi L, Filosa A, Leone L (2016). Testicular sparing surgery in small testis masses: a multi-institutional experience. Arch Ital Urol Androl Organo Uff Soc Ital Ecogr Urol E Nefrol.

[9] Maxwell F, Izard V, Ferlicot S, Rachas A, Correas JM, Benoit G (2016). Color doppler and ultrasound characteristics of testicular Leydig cell tumors. Br J Radiol.

[10] El Sanharawi I, Correas J-M, Glas L, Ferlicot S, Izard V, Ducot B (2016). Non-palpable incidentally found testicular tumors: differentiation between benign, malignant, and burned-out tumors using dynamic contrast-enhanced MRI. Eur J Radiol.

[11] Deshpande SS, Shetty D, Saifi S (2017). Sonographic appearance of testicular adrenal rest tumour in a patient with congenital adrenal hyperplasia. Pol J Radiol.

[12] Keefe DT, Blais A-S, Rickard M, Yehia N, Chami R, Lorenzo AJ (2021). Spermatogenesis in pre-pubertal boys with Leydig cell neoplasms suggests paracrine stimulation by testosterone. J Pediatr Urol.

